# Anther and pollen development: A conserved developmental pathway

**DOI:** 10.1111/jipb.12425

**Published:** 2015-10-22

**Authors:** José Fernández Gómez, Behzad Talle, Zoe A Wilson

**Affiliations:** ^1^ School of BiosciencesUniversity of NottinghamSutton Bonington CampusLoughborough, Leicestershire, LE12 5RDUK

**Keywords:** Anther, *Arabdiopsis*, cereals, comparative biology, pollen

## Abstract

Pollen development is a critical step in plant development that is needed for successful breeding and seed formation. Manipulation of male fertility has proved a useful trait for hybrid breeding and increased crop yield. However, although there is a good understanding developing of the molecular mechanisms of anther and pollen anther development in model species, such as *Arabidopsis* and rice, little is known about the equivalent processes in important crops. Nevertheless the onset of increased genomic information and genetic tools is facilitating translation of information from the models to crops, such as barley and wheat; this will enable increased understanding and manipulation of these pathways for agricultural improvement.

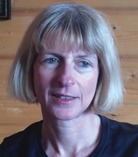

Zoe A Wilson

**Edited by:** Dabing Zhang, Shanghai Jiao Tong University, China

## INTRODUCTION

There are wide‐spread predictions that global populations will reach 9 billion by the end of the next decade (www.fao.org). Maintaining sufficient food for increasing numbers of people is a major world‐wide concern; there is therefore a need to generate increased food production, but to do this in an environmentally sustainable and safe way. This requires not only increased yield, as a consequence of better utilization of resources and productivity, but also decreased losses in production and storage by enhanced resistance to pathogens and pests and by ensuring high quality products. The ability to capture fundamental research and translate this to crops is required to help facilitate improved agriculture to meet the target of global food security. New technologies such as high throughput DNA sequencing and molecular marker analysis, and comparative genomics (e.g., BLAST comparisons) are making the robust identification of orthologous genes possible in economically important species. In addition, the availability of transformation methods, for example *Agrobacterium*‐mediated transformation of rice, wheat and barley, is enabling the characterization of putative orthologous gene functions in species where other resources, such as characterized mutants are scarce.

Feeding an increasing population with less resources and land requires a substantial and constant increase in productivity. Heterosis, or hybrid vigor, has been shown to increase yield by 3.5%–15% (Longin et al. 2012), and has been successfully commercialized in crops such as maize, rice (Zhong et al. 2004; Cheng et al. 2007), barley (Longin et al. 2012) and wheat (Singh 2010). The methods used for hybrid production to ensure cross‐pollination rather than selfing, are diverse and species specific. Approaches include Cytoplasmic Male Sterility (CMS), Chemical Sterility (CHS) or environmental/abiotic sterility (Zhong et al. 2004; Cheng et al. 2007; Longin et al. 2012; Zhang et al. 2013). However, in order to achieve hybrid production, deep understanding of plant fertility and the mechanisms and gene networks that lead to normal pollen formation and release are needed.

Anther and pollen development have been widely studied in *Arabidopsis* (Wilson and Zhang 2009); it is a complex and important process that leads to the release of viable pollen and plant fertilization. The genomic and genetic resources available for the dicot model *Arabidopsis thaliana*, have greatly assisted understanding of this process; however, extensive study of pollen formation has also occurred in rice. Pollen development in both species involves similar key stages (Chen et al. 2005; Itoh et al. 2005); conservation of the regulatory pathways underlying these stages is further demonstrated by the characterization of male sterile mutants from orthologous genes in both species. The application of this information to other species, in particular temperate cereals, will provide opportunities to control fertility in economically important crops such as wheat and barley, in which reproduction is currently less well characterized. The translation of important traits to crops has been greatly assisted by the publication of the complete or partial genome sequences of rice, *Brachypodium*, *Sorghum*, barley, wheat and maize (International Rice Genome Sequencing Project 2005; Paterson et al. 2009; Schnable et al. 2009; International_Brachypodium_Initiative 2010). Syntenic gene identification has provided a very useful approach for ortholog identification (Spannagl et al. 2011). The common ancestry of Angiosperms means that collinearity provides a valuable tool for determining orthlology between taxa, to aid gene annotation and evolutionary analysis. However, genome flexibility and the genome structural evolution provide increasingly greater challenges as the distance between species increases (Spannagl et al. 2011). Nevertheless, syntenic analysis can be complemented by using BLAST/P gene/protein comparisons (Altschul et al. 1990), or by complementation analysis of the model species mutant phenotypes using the candidate orthologous genes (Li et al. 2011; Fernández Gómez and Wilson 2014).

## SYNTENY

Plants and animals evolved independently from unicellular eukaryotes, to form a diverse array of organisms. This diversity in eukaryotic genomes resulted in a large number of non‐coding DNA regions; however, the gene content remained relatively constant (Bennetzen 2000; Jung et al. 2005). Nearly 1,000‐fold variation in genome size is seen between angiosperms, from the 125 Mb genome of *Arabidopsis* to 124,000 Mb genome of *Fritillaria assyriaca*, which is caused at least in part by whole genome duplication (WGD). This WGD motivates comparative approaches that use the available data from the smaller genomes to elucidate the function of genes in the species with larger genomes (Bowers et al. 2003; Dehal and Boore 2005; Tang et al. 2011; Schnable et al. 2012). Chromosomal duplications are widespread and linked with conserved molecular mechanisms, and represent key points in genome evolution (Moore et al. 1995; Salse et al. 2002). However, the occurrence of large gene families poses significant issues for the identification of orthologs in other species; in such cases detailed information on the collinearity of the flanking regions is needed to support ortholog characterization (Salse et al. 2002). Synteny is defined as the preserved order of genes that have originated from a common ancestor and can be used to identify homologous genes in defined regions (Duran et al. 2009). Levels of synteny and collinearity vary depending on the chromosomal region, and reflect underlying eukaryotic genome evolution (Coghlan et al. 2005; Tang et al. 2008). Nevertheless they provide a valuable tool for ortholog characterization (Duran et al. 2009; Spannagl et al. 2011) and analysis of functional relationships between genes (Duran et al. 2009). Comparative mapping studies indicate that most grass species have maintained significant collinearity despite 60 million years of separation (Moore et al. 1995; Salse et al. 2002). This therefore offers a useful approach for comparative analysis between monocots (Xu et al. 2009) and for translation of gene information, underlying agronomically important traits, from model species to economically important crops (Wicker et al. 2001; Duran et al. 2009).

The study of *A. thaliana* has provided substantial understanding relating to characterization of molecular pathways in higher plants, by capitalizing on the available genetic and genomic resources (Spannagl et al. 2011). This knowledge can now be transferred from model systems to economically important species such as wheat, barley, rice, maize (Spannagl et al. 2011), by targeting orthologous genes, with common functions and ancestry (Spannagl et al. 2011).

There has been a major focus for studies into the translational characterization of genes in monocots from characterized genes in the dicot model species *A. thaliana*. Identification and characterization of ZAG1 and ZAG2 in maize is an example of the transfer of knowledge from a model species to a commercially important crop. Schmidt et al. (1993) identified two putative orthologous sequences (*Zea AGAMOUS1*, *ZAG1* and *2*) with high level of similarity at the amino acid level to *Arabidopsis AGAMOUS (AG)* gene. Further analyses showed that the expression of ZAG1 is similar to the *Arabidopsis* gene and is restricted to stamen and carpel primordia (Schmidt et al. 1993). It was also shown that ZAG1 protein binds to putative AG binding sites (Schmidt et al. 1993); a large number of examples are also seen in rice (Table 1) for example functional orthologs of *AG* (*OsMADS53‐58)* (Yamaguchi et al. 2006).

**Table 1:** Anther and pollen development gene network conservation in higher plants

***Arabidopsis***	**Ref**	**Rice**	**Ref**	**Barley**	**Ref**	**Wheat**	**Ref**
**AG**	(1)	OsMADS53‐58	(2)				
**EMS1/TPD1**	(3,4,5)	MSP1/OsTDL1	(6, 7)				
**DYT1**	(8,9)	UDT1	(10)				
**TDF**	(11)						
**AMS**	(12,13,14)	TDR	(13,15,16)				
**MYB103/80**	(17)	OsMYB103/80	(18)				
**MS1**	(19,20,21)	PTC1	(16)	HvMS1	(22)		
**MYB33‐65**	(23)	OsGAMYB	(24)	HvGAMYB	(25)		
**MS2**	(26)	OsDPW	(27)				
**DEX1**	(28)	OsDEX1	(28)	HvDEX1	(28)		
		RAFTIN	(29)			RAFTIN	(29)

(1), Ito et al. 2004; (2), Yamaguchi et al. 2006; (3), Canales, et al. 2002; (4), Canales, et al. 2002; (5), Yang et al. 2003; (6), Nonomura et al. 2003; (7), Zhao et al. 2008; (8), Zhang et al. 2006; (9), Feng et al. 2012; (10), Wang et al. 2006; (11), Zhu et al. 2008; (12), Sorensen et al. 2003; (13), Xu et al. 2010; (14), Xu et al. 2014; (15), Zhang et al. 2011; (16), Li et al. 2006; (17), Zhang et al. 2007; (18), Zhang et al. 2010; (19): Vizcay‐Barrena and Wilson 2006; (20), Yang et al. 2007; (21), Ito et al. 2007a; (22), Fernández Gómez and Wilson 2014; (23), Millar and Gubler 2005; (24), Kaneko et al. 2004; (25), Murray et al. 2003; (26), Aarts et al. 1997; (27), Shi et al. 2011; (28), Ma et al. 2013; (29), Wang et al. 2003. Ref, reference.

## ANTHER AND POLLEN DEVELOPMENT

### Anther development

**Figure 1:**
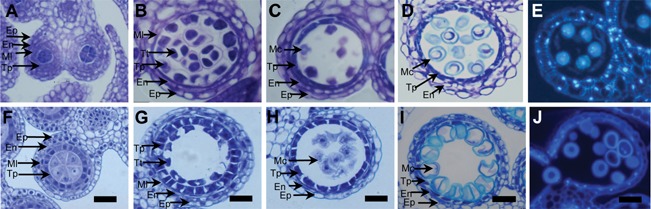
**Anther and pollen development sections of (A–E) *Arabidopsis* and (F–J) barley** Anther and pollen development follows a similar pathway in *Arabidopsis* and barley (**A** and **F**): Secondary sporogenous cells to pollen mother cells. Four cell layers surrounding the anther. (**B** and **G**): Microspore release from the tetrad; the tapetum becomes vacuolated. (**C** and **H**): Free microspores; middle layer becomes crushed and the prominent tapetum starts to degenerate. (**D** and **I**): Microspores become vacuolated and tapetum degenerates. (**E** and **J**): Trinuclear pollen; septum breakage and pollen release. Ep, epidermis; En, endothecium; Mc, microspores; Ml, middle layer; Tp, tapetum; Tt, tetrad. Bars = 50 μm.

Anther development in *Arabidopsis* has been divided into 15 stages, which commence from division of a single archesporial cell; defined cell types and adaxial–abaxial polarity are established, resulting in the formation of the mature microsporangia (Scott et al. 2004). The *Arabidopsis* floral meristem, as for the shoot apical meristem, comprises three cell layers; the stamen primordia are usually initiated by periclinal divisions in the L2 layer (Jenik and Irish 2000). Ultimately these divisions result in an anther comprising four maternal cell layers and an internal layer of sporogenous cells. The initial divisions arise from single L2 archesporial cells, which divide periclinally to form primary parietal cells (PP) subjacent to the L1 and inwardly facing primary sporogenous cell (PS) (Canales et al. 2002). The PS cells undergo a number of divisions to generate the meiocytes, whereas the PP divides periclinally to form an endothecial cell subjacent to the L1 and a secondary parietal cell (SPC). Further periclinal divisions of the SPC generate the middle cell layer, endothecium and tapetum (Scott et al. 2004; Zhang and Yang 2014). In maize, the peripheral L2‐derived (L2‐d) cells undergo asymmetric cell division to generate the endothecium and secondary parietal cell SPC; SPCs then undergo symmetrical divisions to generate the middle and tapetum layers, whereas the centralized L2‐derived cells form sporogenous cells (Kelliher and Walbot 2011). The linear array of different cell types then arises from periclinal divisions of the single archesporial cell. It has been proposed that the sporogenous cells play a key role in organization of the radially symmetrical microsporangium and that a radial field of signals is formed by the PS, inducing periclinal division and development in adjacent cells (Scott et al. 2004). The PP subsequently divides resulting in the endothecium cell and the meristematic SP, which undergo a further division to form the tapetum and middle cell layer (Figure 1).

Anther types are defined based on the divisions of the secondary parietal layers (Carrizo Garcia 2002). Despite similarities in the general pathway of pollen development, four types of anther wall development have been described: Basic (type I), dicotyledonous (type II), monocotyledonous (type III) and reduced (type IV) (Davis 1996). These have tended to be family specific, however some families have more than one type, e.g., Solanaceae (type I and II) (Carrizo Garcia 2002), and the Commelinaceae (type I and III) (Hardy and Stevenson 2000). In the basic type, all SPCs divide periclinally and differentiate to form the four layers, whilst in the dicotyledonous type only the outer SPC layer divides periclinally (Carrizo Garcia 2002), thus the endothecium, middle layers are from the outer SPC, while the tapetum is from the inner SPC. *Brachypodium* presents a typical monocot anther wall type, which is seen in the Poaceae family (Teng et al. 2005), with the outer SPC forming the endothecium, and the ISP layer generating the middle and tapetum layers.

The most conspicuous anther cell layer is the tapetum, which is a single layer of metabolically active cells encasing the developing pollen. The tapetum of spermatophytes are broadly grouped into secretory type, or amoeboid (invasive) types, differing primarily in the extend of their intrusion into the locule during microspore development (Pacini 2010). Amoeboid tapeta intrude into the locule encasing the microspore to provide materials, while the secretory tapetum, such as in *Arabidopsis*, provide nutrients through the liquid in the locule while maintaining their shape (Pacini 2010). The tapetum in *Brachypodium*, is typical of grasses, and is of the secretory‐type (Maheshwari 1950). These cells maintain their position and undergo post‐meiotic degeneration via programmed cell death (PCD) (Papini et al. 1999; Wu and Cheung 2000). The timing of PCD varies between species, for example, in *Brachypodium* and rice degeneration commences at the tetrad stage and is complete by the bicellular pollen stage (Zhang et al. 2011). However, in wheat, breakdown of tapetum cells appears to begin during the vacuolated microspore stage (Mizelle et al. 1989), equivalent to that observed in barley (Figure 1) (Fernández Gómez and Wilson 2014). The degeneration of the tapetum is required for release of pollen wall materials onto the developing pollen (Pacini 2010), but is also important for normal dehiscence of the anther.

One principal characteristic of secretory tapeta in grasses is the production of spheroid electron‐dense structures (orbicules/Ubisch bodies). Ubisch bodies are granules of sporopollenin lining the inner tangential and sometimes the radial walls of tapetum cells (Heslop‐Harrison 1968; Quilichini et al. 2014). Studies on Ubisch body formation in *Brachypodium* support published evidence that they are formed in the tapetum and are involved in exine synthesis (Sharma et al. 2014), however, their function has not been fully established. In barley, numerous Ubisch bodies (Radchuk et al. 2012), are associated with the secretory tapetum and surround the microspores (Parish and Li 2010). This secretion continues until the tapetum has completely disintegrated. The rice tapetum also produces characteristic orbicules/Ubisch bodies that export sporopollenin precursors to the locule (Zhang et al. 2011), whereas in wheat pre‐Ubisch bodies, are produced in the tapetum cells and form the core of Ubisch bodies onto which sporopollenin is deposited (El‐Ghazaly and Huysmans 2001). Orbicules have not been observed in *Arabidopsis* or other Brassicaceae, however, secretory organelles, such as elaioplasts and tapetosomes are seen (Wu et al. 1997). Little is known about Ubisch bodies, however, a rice and wheat anther‐specific gene, RAFTIN, has been identified in pro‐orbicule bodies and shown to accumulate in Ubisch bodies (Wang et al. 2003). RAFTIN is targeted to the microspore exine and is critical for late pollen development in cereals (Wang et al. 2003); no dicot homolog has been identified in *Arabidopsis*.

### Pollen development

**Figure 2:**
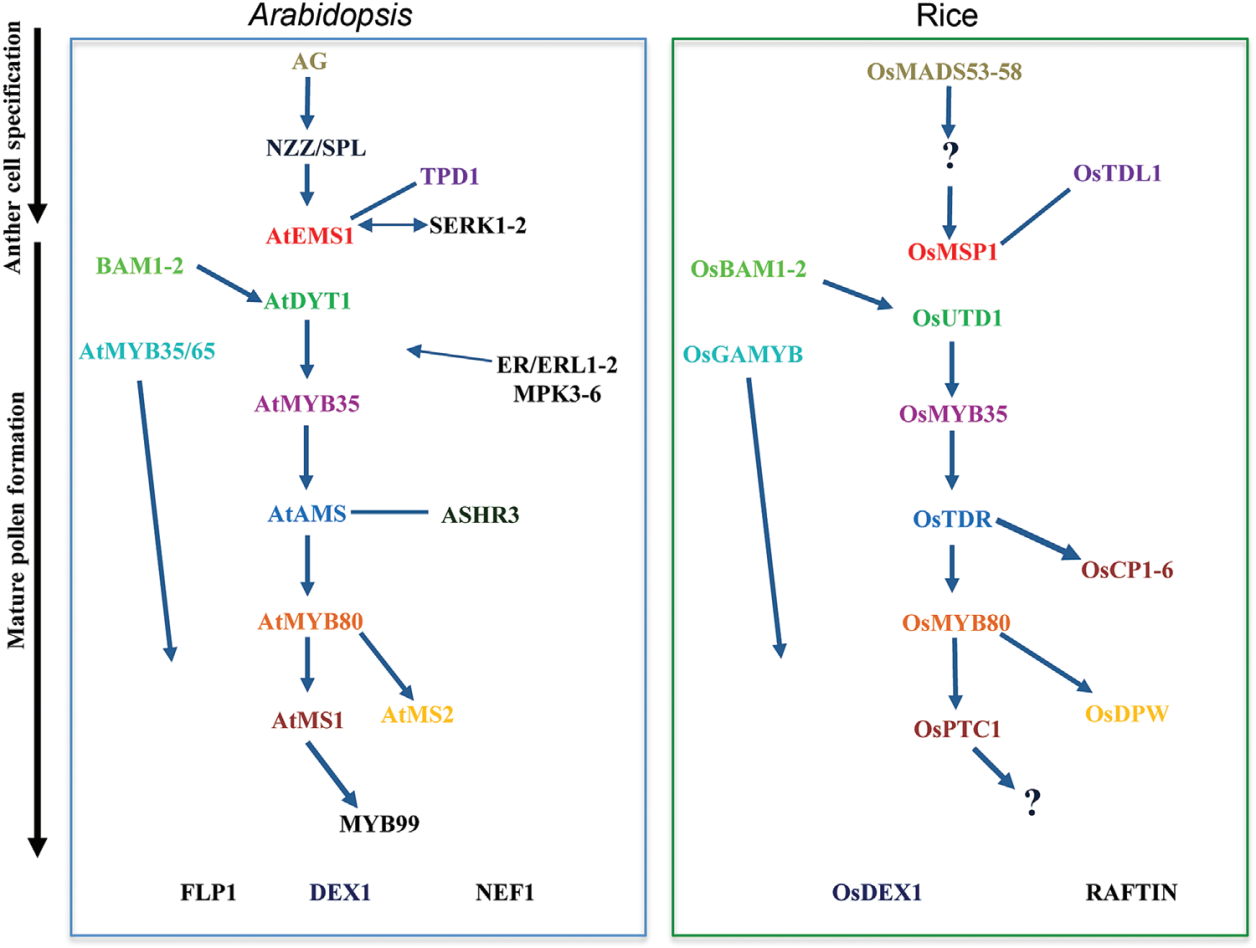
**Anther and pollen development gene regulation network for *Arabidopsis* and rice from anther cell specification to mature pollen formation** Comparisons between species show similarities in the regulation of pollen development; unconnected regions and “?” indicate where regulatory network is still undefined. Colors correspond to equivalent orthologs.

Pollen development comprises three major stages: (i) microsporogenesis (differentiation of the sporogenous cells and meiosis); (ii) post‐meiotic development of microspores; and (iii) microspore mitosis (Chaudhury 1993) (Figure 2). Sporogenous cells (pollen mother cells or meiocytes) are encased in a tapetum‐derived callose wall and undergo meiosis to form tetrads of haploid microspores (Owen and Makaroff 1995). This callose layer appears essential for development and formation of the scaffold for the pollen wall, it then breaks down and the microspores are released into the anther locule to begin male gametophyte development (post‐meiotic development). The free micropsores then go through one/two rounds of mitotic divisions and pollen wall formation continues. The final mature pollen may be tricellular, or as seen in some species it can be bicellular with the final mitotic division occurring in the pollen tube. The anther filaments elongate rapidly and the flowers and anthers then open (stage 13), enabling effective release of the pollen. The sporophytic anther tissues, in particular the tapetum cell layer, play an essential part in this developmental process both in the regulation and coordination of development, but also in providing materials for pollen wall formation.

During pollen mitosis I, the microspore divides asymmetrically to produce a vegetative and a generative cell. Following the first mitotic division, a second mitosis produces two sperm cells enabling double fertilization to produce the embryo and endosperm (Eady et al. 1995). This asymmetric division is essential for the establishment of male germ cells and symmetrical division of the microspore fails to generate germ cells (Eady et al. 1995). Asymmetrical division is dependent on microtubules and most of the mutants described that affect this division are microtubule‐related (Twell 2011). For example, *gemini pollen1* and *two‐in‐one*, both fail in cytokinesis (Twell et al. 2002; Oh et al. 2005), and TUBG1 and 2 are required for spindle and polar division of the microspore (Pastuglia et al. 2006). Although asymmetrical division has been shown to be essential for germ cell formation, the controlling mechanisms for this are currently unknown (Twell 2011).

Sperm cell are transcriptionally active and produce a wide diversity of transcripts (Singh et al. 2008). A number of sperm cell genes have been characterized as essential for fertilization, among them, HAP2/GCS1, a sperm‐specific surfaced linked protein required for fertilization and pollen tube signaling (von Besser et al. 2006; Frank and Johnson 2009) and SHORT SUSPENSOR (SSP), a protein that triggers the asymmetrical division of the zygote (Bayer et al. 2009). Sperm cell mRNA accumulates and is stored for use during germination and pollen tube growth. RNA accumulation varies between species, in addition, the types of RNAs (RNAt, RNAr and RNAm) peak at different stages depending on the species (Mascarenhas 1990). A high number of new transcripts have been detected in mature pollen of *Lilium* and tobacco; similar accumulation of novel transcripts is also seen in maize and tomato (Stinson et al. 1987; Twell et al. 1989).

Early transcriptome analysis in *Arabidopsis* (Becker et al. 2003; Lee and Lee 2003; Honys and Twell 2004) indicated between 3,500 and 5,500 pollen‐expressed genes (Rutley and Twell 2015). The nature of these genes varies depending on the species. For instance, in *Arabidopsis*, genes involved in signaling, cell wall metabolism and cytoskeleton are enriched, indicating the importance of stored mRNA after pollination, tube growth and signaling for pollen–pistil interactions. Further studies have indicated between 3,954 and 7,235 genes are expressed in mature pollen (Honys and Twell 2004; Pina et al. 2005; Schmid et al. 2005; Borges et al. 2008; Wang et al. 2008; Qin et al. 2009).

As in *Arabidopsis*, rice shows a similar high number of genes expressed in mature and germinated pollen (5,939 and 5,945 respectively); their pattern of expression is similar; however, differences are observed in stage‐specific expression. For example, rice expresses more transcripts associated with defense and stress responses in mature pollen. Many transcription factors have been identified, and these are shared in *Arabidopsis* and rice. For instance, the MIKC*MADS‐box network, a transcription factor family required for pollen maturation (Liu et al. 2013). Studies have shown five pollen‐specific MIKC*MADS boxes proteins in *Arabidopsis* and three in rice, S‐(OsMADS62, OsMADS63) and P‐(OsMADS68) (Verelst et al. 2007a, 2007b). Similar phenotypes have been observed in *Arabidopsis* and rice mutants, where pollen germination failure and reduced pollen viability, or abnormal starch accumulation have been seen suggesting a conserved regulatory network, nevertheless unique components of this network have also been observed (Liu et al. 2013). Differences have been also seen in tobacco in relation to the decline of transcripts from microspores to mature pollen, this may reflect different demands in tobacco that explain the temporal shift in the peak of maximum transcription (Bokvaj et al. 2015).

The MYB transcription factor DUO1 has been shown to be essential for germ cell differentiation and fertilization, and is involved in the upregulation of at least 63 germ‐line specific genes (Borg et al. 2011). DUO1 directly regulates three genes, GCS1/HAP2 (Liu et al. 2008; Steele and Dana 2009; Wong and Johnson 2010), GEX2 that contribute to the accumulation of CYCB1;1 (Brownfield et al. 2009) and therefore to the progression through the cell cycle G2/M phase, and MGH3 (HTR10) (Okada et al. 2005; Ingouff et al. 2007), a male germline‐specific histone that functions by chromatin‐binding to regulate transcription (Russell et al. 2012). This pathway seems to be highly conserved in rice where three close homologs to *Arabidopsis* H3, TRT704, HRT11 and HRT12, are highly transcribed in germ cells (Russell et al. 2012). In addition, the expression of DAZ1 and DAZ2, two Ethylene Response Factor‐associated ampliphilic repression (EAR) motif proteins are DUO1‐dependant in sperm cells (Borg et al. 2011). DAZ1 and 2 are required for germ cells to enter mitosis and for accumulation of mitotic cyclins. In addition, they are also necessary for the expression of genes involved in germ‐line differentiation, and therefore for successful fertilization (Borg et al. 2014). In the *duo1* mutant, *DAZ1* and *2* are suppressed, which also affects the downstream germ‐specific DUO1 targets such as HTR10/MGH3 (Borg et al. 2011). The *duo1*mutant germ cells fail to enter mitosis; however, *DAZ1* expression can restore mitotic division, whereas deficient *daz1‐2* germ cells that fail to enter mitotic division cannot be rescued by normal expression of *DUO1* (Borg et al. 2014). DUO1 transcriptional regulation also appears conserved in rice sperm cells (Russell et al. 2012). Other sperm genes, such as aquaporins, F‐box motif proteins, or ubiquitin pathway related proteins are highly transcribed and generally appear to be conserved between monocots and dicots plants (Russell et al. 2012).

### Pollen wall formation

The tapetum helps provide essential materials for pollen wall formation and the regulation of development for the microspores during PMC meiosis and subsequent microspore and pollen maturation. The tapetum plays an active role in this process being responsible for the callose secretion, breakdown and synthesis of many pollen wall materials (Xu et al. 2014). Callose deposition is essential for the early stages of the exine formation, with aberrant deposition/breakdown frequently resulting in a failure of viable pollen development (Chasan 1992; Dong et al. 2005). Pollen wall formation is initiated while the callose wall is in place; the primexine, a microfibrillar polysaccharide matrix, is formed by the microspores this serves as the pattern for the final sculptured pollen wall (exine) (Owen and Makaroff 1995). The exine is predominantly composed of sporopollenin, this is secreted by the tapetum and polymerizes onto the primexine, providing anchoring sites for baculae formation. The callose wall is essential in this, as indicated by the callose synthase (*cals5*) mutant, which forms a defective exine, with the globular structures rather than defined baculae and tectum (Dong et al. 2005). Primexine deposition is also essential for pollen viability. *ARF17*, an auxin responsive factor, plays a major role in plant fertility by regulating primexine deposition. The *arf17* mutant shows defective callose formation, a total absence of primexine and pollen degeneration (Yang et al. 2013). In addition, it has been demonstrated that ARF17 directly regulates CalS5expression (Yang et al. 2013). *CalS5*expression is also reduced in the *defective in exine formation1* (*dex1)* mutant; this gene encodes for a plasma membrane protein, which is highly conserved in higher plants such as rice, barley and *Brachypodium* (Ma et al. 2013) (Table 1). The *dex1* mutant shows defective pollen wall formation from microspore release, resulting in microspore abortion by stage 9. *DEX1* therefore appears essential for the early stages of exine formation and sporopollenin secretion (Ma et al. 2013).

After meiosis, the tapetum secretes β1,3 glucanase (callase), which breaks down the callose layer surrounding the tetrads and releasing the microspores into the locule (Lu et al. 2014). The timing of callase secretion appears critical for pollen development (Bedinger 1992), with miss‐expression causing sterility (Worrall et al. 1992). A low level of activity is present during the first meiotic division, but once the second meiotic division takes place, this rapidly increases and peaks at the time of microspore release (Chasan 1992; Worrall et al. 1992).

Pollen wall deposition is regulated in part by the tapetum, but also by the developing microspores/pollen. Osmiophilic lipid bodies accumulate in the tapetum, these are exported by exocytosis into the locule; polymerization of the acyl precursors forms the sculptured exine wall (Yang et al. 2007). The inner, tangential and radial surfaces of the tapetum appear to have secretory properties; vesicles fuse to the radial and tangential membranes, releasing their contents into the anther locule. The tapetal cells begin to accumulate elaioplasts and large cytoplasmic lipid bodies, and to become increasingly vacuolated. After the first pollen mitotic division, the tapetum goes through regulated breakdown via programmed cell death (PCD); this enables release of tapetum materials into the locule for deposition onto the developing pollen grains (Vizcay‐Barrena and Wilson 2006; Parish and Li 2010). This material, “pollenkitt” and tryphine, fills the gaps between the baculae to form the pollen coat (Owen and Makaroff 1995).

Despite the great diversity of pollen surface morphology, spore/pollen walls exhibit common structural features (Wallace et al. 2011). These typically include an inner intine composed of pectin, cellulose, and hemicellulose, and an outer exine composed of sporopollenin (Heslop‐Harrison 1968; Quilichini et al. 2014). In addition, for many species, including *Arabidopsis*, this structured backbone is covered by a heterogeneous pollen coat called tryphine, which is involved in pollen stigma adhesion, recognition and hydration (Piffanelli et al. 1998; Edlund et al. 2004; Murphy 2006). Intine secretion is regulated by the microspores, although the maternal anther tissues may also be involved in the control of this process. The intine is composed of two layers, the external‐facing granular exintine, and a microfibrillar endintine. Outside of this is the exine, comprising nexine and the outer sculpted sexine, which consists of tectum and baculae. The exine is composed predominantly of sporopollenin, which consists of fatty acid derivatives and phenolic compounds (Piffanelli et al. 1998; Blackmore et al. 2007). However, recent investigations suggest that the nexine is composed of arabinogalactan proteins, these proteins contain a large arabinogalactan polysaccharide chain and arabino oligosaccharides (Ellis et al. 2010). Nexine formation appears regulated by the Tapetal AHL family protein (TEK), an AT‐Hook protein that is expressed in the tapetum during the tetrad stage; in the *tek* mutant nexine formation is absent (Lou et al. 2014). Four AGPs arabinogalactan proteins, AGP6, AGP11, AGP23 and AGP40, are upregulated by TEK, and are thought to form the backbone of the nexine layer (Jia et al. 2015). Expression of *AGP6* in this mutant can partially restore nexine formation and plant fertility (Jia et al. 2015).

Sporopollenin is extremely resistant and provides the pollen grains with effective protection from environmental stress (Meuter‐Gerhards et al. 1999). This resilience to degradation has meant that it has been difficult to determine its exact composition. Recent studies using extant pollen suggest it comprises primarily oxygenated, aromatic monomers, particularly ferulic and p‐coumaric acids (de Leeuw et al. 2006); fossilized sporopollenin has been shown to have a higher aliphatic content, this may be due to biosynthetic differences, treatment/fossilization (de Leeuw et al. 2006; Ariizumi and Toriyama 2011). Sporopollenin composition, structure and biosynthesis appear conserved among all land plants. Phylogenetic and genomic analysis support this as putative orthologs of *ACOS5*, *PKSA*, *PKSB*, *CYP703A2* and *CYP704B1* are present in flowering plants, but absent from green algae (Wang et al. 2003; de Azevedo Souza et al. 2009; Kim et al. 2010; Wallace et al. 2011; Yang et al. 2014). Supporting this hypothesis, *Physcomitrella patens* encodes for an enzyme with *in vitro* preference for hydroxyl fatty acyl‐CoA esters that is capable of hydroxyalkylpyrone synthase activity, suggesting that *PpASCL* is a functional ortholog of *Arabidopsis PKSA* and that the pathway to sporopollenin is conserved in land plants (Colpitts et al. [Link]). In addition, the rice *OsCYP704B2* that encodes a long chain fatty acid is capable of metabolizing similar substrates *in vitro* as *Arabidopsis CYP704B1* (Li et al. 2010). A similar function was observed for *Arabidopsis MS2* (Aarts et al. 1997) and the rice orthologs, *OsDPW* (*DEFECTIVE POLLEN WALL*); *DPW* can restore exine formation in the *ms2* mutant, further supporting the conservation of sporopollenin synthesis among monocotyledonous and dicotyledonous plants (Shi et al. 2011).

### Regulation of anther and pollen development

Numerous regulatory genes have been identified in *Arabidopsis* as responsible for anther cell differentiation and pollen formation (Figure 2). Access to genome sequence, mutants and efficient *Agrobacterium*‐mediated transformation in species such as rice and barley are allowing the characterization of orthologous genes involved in anther and pollen development in *Arabidopsis* (Figure 2; Table 1) (Wilson and Zhang 2009). Although some genes appear to be species‐specific, pollen development in higher plants seems to be a highly conserved process (Figure 2).

The *SPOROCYTELESS/NOZZL*E (*SPL/NZZ*) transcription factor (Schiefthaler et al. 1999; Yang et al. 1999) is one of the early acting regulators that is required for the initiation of cell division and differentiation in stamen and carpels. It is induced by the MADS box transcription factor AGAMOUS (AG) (Ito et al. 2004). AG shows prolonged expression, with early roles in floral initiation via stamen and carpel specification (Ito et al. 2004) and during later development via induction of *DEFECTIVE IN ANTHER DEHISCENCE1* (*DAD1*) (Ito et al. 2007b) which catalyses jasmonic acid (JA) biosynthesis and results in stamen filament extension and flower opening. AG function is conserved in rice and associated with *OsMADS53* and *OsMADS58* (Yamaguchi et al. 2006) (Figure 2; Table 1).

NZZ/SPL acts in the anther L2 cell layer during archesporial division. In the mutant, formation of pollen mother cells (PMCs) and the surrounding cell layers fails (Schiefthaler et al. 1999; Yang et al. 1999), suggesting that SPL/NZZ determines anther and ovule development via interaction with the surrounding cell layers. In maize, the *MULTIPLE ARCHESPORIAL CELLS1* (*MAC1)* gene acts on archesporial cells and thus appears to function earlier than SPL/NZZ. The *mac1* mutant produces multiple archesporial cells in the ovule (Sheridan et al. 1996), while in the anther archesporial specification is normal but causes meiotic arrest to occur. Thus *SPL/NZZ* appears to act upstream of *MAC1* in anthers (Yang et al. 1999).

The early steps linked to archesporial cell division in the L2 layer and the control of the numbers of cells that will form sporogenous initials are regulated by *EXTRA SPOROGENOUS CELLS/EXS MICROSPOROCYTES*1 (*EXS/EMS1*) (Canales et al. 2002; Zhao et al. 2002), and *TAPETAL DETERMINANT1* (*TPD1*) (Yang et al. 2003). In the *exs1/ems1* mutant, the tapetal and middle cell layer are absent and replaced with additional meiocytes. *EXS1/EMS1* encodes for a putative serine threonine leucine rich repeat (LRR) receptor kinase (Canales et al. 2002; Zhao et al. 2002), which acts by complexing with TPD, a small protein, which serves as a ligand for EMS1 (Yang et al. 2005; Jia et al. 2008). This serves to restrict differentiation in the L2 layer of the anther, or enhance embryo growth (Canales et al. 2002). Both *ems1* and *tpd1* mutants are male sterile with a block in development during meiosis II, suggesting that although initiation of meiosis is possible a functional tapetum is needed for the later stages of meiosis and on‐going pollen development (Yang et al. 2003).

Early anther specification also involves a number of leucine‐rich repeat receptor‐like protein kinases (LRR‐RLKs), including BAM1 and BAM2 (BARELY ANY MERISTEM) (Hord et al. 2006). BAM1/BAM2 act redundantly to control differentiation of parietal cells for formation of the tapetum, middle cell and endothecium layers. In the double mutant these cells are missing although PMC‐like cells are seen; however, these subsequently degenerate, suggesting that BAM1/BAM2 controls sporogenous cell number by promoting differentiation of the surrounding somatic cells (Hord et al. 2006). BAM1/BAM2 function appears conserved in other species, since homologs have been identified in poplar (*Populus trichocarpa*) and rice (*Oryza sativa*) (Figure 2).

A number of other LRR‐RLKs, including the redundant Somatic Embryogenesis Receptor‐Like Kinase 1 (SERK1 and SERK2), ER family of LRR‐RLKs (ER, ERL1 and ERL2) and MPK3‐6, are also involved in early tapetum development and cell differentiation (Albrecht et al. 2005; Colcombet et al. 2005; Hord et al. 2008). The *serk1‐2* double mutant phenotype is similar to the *ems1/exs* and *tpd1* mutants, with an absence of the tapetum and increased sporocytes. The *er/erl1/erl2* mutants have abnormal cell patterning, with increased numbers of tapetum and occasionally middle layers cells, indicating that they are involved in regulating tapetum differentiation signals (Hord et al. 2008). *MPK3/MPK6* also impacts upon anther differentiation; however although both the *er/erl1/erl2* and *mpk3/mpk6* mutants have similar phenotypes to *ems1/exs*, *tpd1* and *serk1/ 2* they produce a tapetum. Expression of *EMS1* and *TPD1* is not affected in the *mpk3/6* mutant, suggesting that they act via an independent pathway (Hord et al. 2008).

In rice, EMS1 and TPD1 orthologs, MULTIPLE SPOROCYTE (MSP1) and OsTDL1/MIL2 (MICROSPORELES 2) (Figure 2; Table 1) have conserved and diversified roles (Nonomura et al. 2003; Zhao et al. 2008). A similar phenotype to that of *ems1/exs* and *tpd1* is seen in the *msp1* mutant; however, MIL2 transcript and protein are expressed in the inner parietal cells, which differs from TPD1. In addition, *mil2* showed two anther wall layers, suggesting a key role in specifying the differentiation of primary parietal cells in rice (Hong et al. 2012). These differences indicate that EMS1/EXS‐TPD1 signaling varies among species during reproduction. For instance, the maize *TPD1* homolog, *MULTIPLE ARCHESPORIAL CELLS1* (*MAC1*), in contrast to its *Arabidopsis* homolog, is essential for supressing archesporial cells proliferation and promoting periclinal division of sub epidermal cells. MAC1 also differs from *Arabidopsis* TPD1 and rice MIL2, in its localization in archesporial cells, its secretory protein nature, and the absence of a role in epidermal function and meiotic cell specification (Wang et al. 2012).

DYT1 is a basic helix‐loop‐helix (bHLH) transcription factor that acts downstream of *SPL/NZZ* and *EMS1/EXS*. Anther cell specification is normal in the *dyt1* mutant; however, during meiosis the tapetum develops enlarged vacuoles and microspore degeneration subsequently occurs (Zhang et al. 2006). DYT1 appears to function in meiotic progression alongside the regulation of many tapetal genes (Feng et al. 2012). The rice ortholog, *UNDEVELOPED TAPETUM1* (*UDT1*) (Figure 2; Table 1), appears to act in a similar way to *AtDYT1* (Jung et al. 2005). The expression of both *UDT1* and *DYT1* is reduced in the corresponding *msp1* and *ems1/exs* mutants (Wang et al. 2006) suggesting conserved regulation of these transcription factors in *Arabidopsis and* rice (Figure 2; Table 1).

*TAPETAL DEVELOPMENT and FUNCTION (TDF1/MYB35)* gene is also an essential regulator for tapetum development and function. It acts downstream of DYT1 and upstream of *ABORTED MICROSPORES (AMS)* (Zhu et al. 2008, 2011). Recent investigation shows that DYT1 directly regulates *TDF1* in the tapetum. The expression of TDF1 in the *dyt1* mutant can restore the expression of downstream genes including *AMS*, *MS188*, *TEK* and other sporopollenin genes (Gu et al. 2014). The *AMS* gene encodes for a putative bHLH‐type transcription factor, which has a low level of anther expression pre‐meiotically and then increases post‐meiosis (Sorensen et al. 2003). AMS acts as a master regulator of tapetum gene expression associated with tapetum function and biosynthesis, including the synthesis of lipidic and phenolic components that are essential for pollen wall pattering, and flavonoids (Xu et al. 2010, 2014). The *ams* mutant presents premature microspore degeneration due to the reduced callose wall and an absence of sporopollenin secretion (Xu et al. 2014), the tapetum becomes abnormally enlarged and vacuolated (Sorensen et al. 2003). The *Arabidopsis* SET‐domain protein, ASHR3, has been shown to interact with AMS, implying that ASHR3 may target AMS to chromatin to regulate stamen development (Thorstensen et al. 2008).

An *AMS* ortholog has been described in rice, *TAPETUM DEGENERATION RETARDATION* (*TDR*) (Xu et al. 2010) (Figure 2; Table 1). *OsTDR* shows an equivalent expression pattern to *AMS*; *tdr* mutants have normal development except for pollen degeneration and male sterility, due to defects in tapetum function and PCD (Zhang et al. 2011). TDR regulates tapetum degradation at least in part via interaction with ETERNAL TAPETUM 1 (*EAT1)* a bHLH transcription factor, which positively regulates two aspartyl proteases OsAP25 and OsAP37 that are involved in directing tapetum PCD (Niu et al. 2013). The *MYB103/MS188* transcription factor is also important for tapetum development and function, with mutants showing early tapetum degeneration and microspores with defective exine and pollen coat that ultimately abort (Zhang et al. 2007). AtMYB103 directly downregulates VGD1 and GLOX1, and upregulates A1 (UNDEAD) (Phan et al. 2011). A1 is an aspartic protease that shows premature tapetal and pollen degeneration in a similar phenotype to the *myb80* mutant. AtMYB80 binds CIS elements of the promoters of these three genes, which all show a similar core sequence, AACC. MYB80/UNDEAD regulates PCD timing, as indicated by enhanced DNA fragmentation, as detected by TUNEL assays (Phan et al. 2011).

The *AtMYB103* rice ortholog, *OsMYB103* (Figure 2; Table 1), has a similar expression pattern and function in rice (Zhang et al. 2010). AtMYB103 is essential for expression of MALE STERILITY2(MS2) (Zhang et al. 2007), a Fatty Acyl Carrier Protein Reductase which regulates exine formation in *Arabidopsis*. This interaction has also been observed in rice where the *OsMS2* (*OsDPW*) is downregulated when *OsMYB103* expression is reduced (Zhang et al. 2010), confirming the anther and pollen regulation network also at this level.

MALE STERILITY 1 (MS1) is Plant Homeodomain (PHD)‐finger transcription factor that is crucial for pollen wall formation and tapetum PCD (Vizcay‐Barrena and Wilson 2006; Yang et al. 2007). Orthologs of this gene have been found in rice (*OsPTC*) (Li et al. 2011) and barley (*HvMS1*) (Fernández Gómez and Wilson 2014) (Figure 2; Table 1). MS1 shows very transient expression in the tapetum, from callose breakdown to the free microspore stage (Yang et al. 2007). The PHD‐finger motif is conserved in plants, yeast and human, which is present in histone methyltransferase, histone acetyltransferase and DNA binding proteins, and has been linked with chromatin remodeling (Wilson et al. 2001; Ito et al. 2007a).

**Figure 3:**
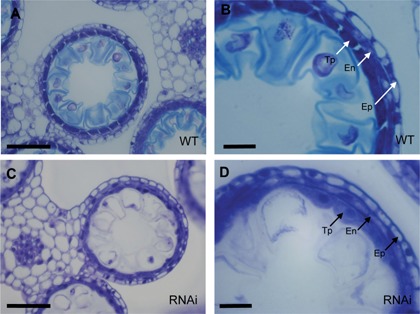
**Transverse sections through barley anthers of sterile *HvMS1*‐RNAi silencing lines and wild type (WT)** (**A**, **B**) Section of a wild type anther at late free microspore stage. Middle layer has disappeared but the tapetum is still intact. (**C**, **D**) *HvMS1*‐RNAi lines at free microspore stage. Silencing lines showed early tapetum degeneration when compared to the wild type (B). Ep, epidermis; En, endothecium; Tp, tapetum. Bar: A and C: 50 μm, B and D: 20 μm.

In the *ms1* mutant, degeneration of immature pollen occurs soon after microspore release; the tapetum becomes abnormally vacuolated with altered degeneration and the microspores appear sticky with minimal exine formation, suggesting an unusual pollen wall composition (Vizcay‐Barrena and Wilson 2006; Ito et al. 2007a; Ariizumi and Toriyama 2011). Histological analysis of the rice *ptc1* mutant indicates a similar phenotype to *ms1* with a failure of tapetum PCD; however, an additional over‐proliferation of the tapetum with an abundance of organelles is seen. The tapetum cytoplasm becomes extruded into the locule; however, this remains constrained by the plasma membrane (Li et al. 2011). Expression of *PTC1* was not reduced in the *utd1* or *tdr* mutants (Jung et al. 2005), but was significantly lower in *gamyb‐2* (Aya et al. 2009), suggesting that GAMYB may be upstream of PTC1 in rice. In barley, *HvMS1*RNAi silencing led to complete sterility in some tillers, alongside occasional normal, fertile spikes, with sterile spikes showing premature degeneration of the tapetum at the free microspores stage (Figure 3) (Fernández Gómez and Wilson 2014). In addition, *HvMS1* overexpression caused complete male sterility with failed dehiscence, residual tapetum and sticky pollen (Fernández Gómez and Wilson 2014). This agrees with previous reports in *AtMS1* overexpression lines, which exhibited expanded and uneven epidermal anther cells, and sticky pollen, suggesting an abnormal pollen wall (Yang et al. 2007).

The anther and pollen gene development network comprises of a complex system of gene expression and interactions; however, although species may have some unique mechanisms, there is a considerable degree of conservation (Wilson and Zhang 2009). The morphology of anther development and the pollen formation are remarkably similar between mono and dicot plants (Figure 1), suggesting that equivalent interactions are occurring at the molecular and genetic level. Nevertheless, only a small number of genes have been shown to have conserved functions between mono and dicots. This may be principally due to the lack of sequence information in species such as wheat and barley, and difficulties in characterizing the candidate genes. However, a number of bioinformatics and molecular tools are now available and progress in revealing the anther and pollen development network across to temperate cereals, including wheat and barley should be swifter.

## NEW TOOLS FOR TRANSLATION ANALYSIS

Translation of information from the pollen development regulatory network from models systems such as *Arabidopsis*, to temperate cereals including wheat and barley has been limited by a lack of genomic resources. Nevertheless recent advances, such as the release of fully annotated genomic sequence data are enabling rapid progress in this area; five grass genomes are now available (*Hordeum vulgare*, *Oryza sativa*, *Sorghum bicolor*, *Brachypodium distachyon* and *Zea mays B73*) (International Rice Genome Sequencing Project 2005; Paterson et al. 2009; Schnable et al. 2009; Vogel 2010; Mayer et al. 2012). Bioinformatics tools and databases have proliferated and can be used to find and compare homologous sequences, for example Gramene and TAIR (www.gramene.org and www.Arabidopsis.org) are valuable for finding and comparative functional genomics for *Arabidopsis*, rice, barley and *Brachypodium* DNA and protein sequences. Gramene (http://www.gramene.org) provides access to 27 fully and 10 partially sequenced reference genomes, and enables the use of ontologies to integrate structural and functional annotation data. Comparative genomic alignments, combined with phylogenetic gene family trees can then be used to infer syntenic and orthologous relationships. Gramene ortholog analysis offers information about the main models and grasses including *Arabidopsis*, rice, *Brachypodium*, barley, *Sorghum* and poplar. Although these orthologs are not always accurate, and extra analysis must be conducted, this preliminary information is of great value. The site also includes genome mapping for 10 species, a pathway section, where pathways, reaction and metabolites can be analyzed and marker and QTL map information.

Later releases such as the IPK Barley Blast Service (http://webblast.ipk-gatersleben.de/barley/) (Mayer et al. 2012), or the International Wheat Genome Sequencing Consortium (IWGSC), also provide platforms for gene discovery and understanding the fundamental biology underlying traits for crop improvement. The International Barley Consortium provides an integrated resource for physical, genetic and functional sequence analysis, enabling analysis in a whole‐genome context. A physical map of 4.98 Gb has been developed, comprising >3.9 Gb of sequence that is anchored to a high‐resolution genetic map. Shot‐gun sequencing, combined with cDNA and RNA sequence data has been aligned onto this framework to generate 79,379 transcript clusters comprising of approx. 26,000 genes that are supported by homology analyses to other species. The extent of this resource means that barley serves as an effective model for the Triticeae, which also includes durum and bread wheats, and rye.

In 2005 a group of scientists, growers, and public and private breeders established the International Wheat Genome Sequencing Consortium (IWGSC), with the aim of enhancing understanding of the wheat genome by sequencing. The goal of IWGSC is to make sequence data and the associated DNA‐based tools freely available. The Wheat Portal (http://wheat-urgi.versailles.inra.fr/Seq-Repository/BLAST) provides access to sequence information genetic and physical maps, markers, phenotypes and array data; it also enables access to analysis tools such as annotation pipelines. This is supported by URGI at Institut National de la Recherche Agronomique (INRA), which is a plant and crop parasite genomics and bioinformatics research centre.

Basic Local Alignment Search Tool (BLAST) (Altschul et al. 1990) analysis has become an essential tool for any comparative bioinformatics analysis to identify regions of similarity between nucleotide or protein sequences, which can infer evolutionary relationships, and help functional analysis and gene family characterization. BLAST analysis can be carried out on most species‐specific databases; however, the National Center for Biotechnology Information (NCBI; www.ncbi.nlm.nih.gov/guide) provides a valuable centralized resource for genomic research by integrating data from >20 biological databases with almost 12 million plant‐derived sequences, comprising 160,000 organisms and approximately 60,000 plant taxa; these data can be mined using the Entrez search and retrieval system. The service offers a wide range of options that are very useful. For instance, the Needleman‐Wunsch Global Align Nucleotide/amino acids Sequences Alignments may be classified as either global or local. Local alignments algorithms (such as BLAST) are most often used (Altschul et al. 1990), while global alignment can be used where significant similarity is expected over their entirety, in which case this may return a better presentation.

**Figure 4:**
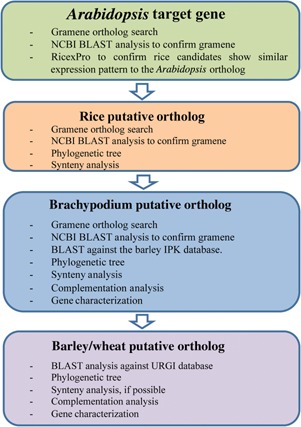
**Diagram showing the various steps to identify putative orthologous genes in barley and wheat starting from an *Arabidopsis* gene** Several bioinformatics tools are available to compare/find sequences, analyze expression patterns, establish phylogenetic trees or synteny analysis. URL; Gramene: http://www.gramene.org/; NCBI: http://blast.ncbi.nlm.nih.gov/Blast.cgi; RicexPro: http://ricexpro.dna.affrc.go.jp/; URGI: http://wheat-urgi.versailles.inra.fr/Seq-Repository/BLAST; Barley IPK database: http://webblast.ipk-gatersleben.de/barley/.

Translational research requires the utilization of model plant genes and the comparison between these gene sequences and the databases of the species where orthologs may be found. However, finding ortholog genes in wheat and barley requires model plant databases where similarities are higher (Figure 4). The rice and *Brachypodium* genomes (Eckardt 2000; International Rice Genome Sequencing Project 2005; Vogel 2010) provide valuable and highly complete information. Many genes have been localized in rice using *Arabidopsis* sequences and their characterization indicated their homology and functional conservation in anther and pollen development (Wilson and Zhang 2009). BLAST analysis between rice genes and barley databases has been useful to localize ortholog genes; however, given the evolutionary distance between *Arabidopsis* and the temperate cereals, *Brachypodium* has been valuable as a bridge between the characterized *Arabidopsis*/rice genes and barley (www.Brachypodium.org). Rice and *Brachypodium* sequences can be generally BLAST analyzed against the IPK viroblast database (http://webblast.ipk-gatersleben.de/barley/) and the sequences found then analyzed by BLAST against the *Hordeum vulgare* NCBI database, to obtain accession numbers and confirm the previous results. In addition, accession numbers and the sequences can be compared against those defined as orthologs in the Gramene database. Phylogenetic trees (Fast Minimum Evolution Method) (Desper and Gascuel 2004) can be used to determine the closest sequences amongst those that result from the BLAST analysis.

Finding ortholog sequences in barley when no rice ortholog has been characterized requires a different approach. BLAST between *Arabidopsis* candidates and rice database generally produces very low similarities results, normally no higher than 50%. Therefore, from the number of sequences that are considered the closest, several analyses have to be performed (Figure 4). Expression analysis is a very useful approach, as the genes associated with pollen development have highly specific expression patterns, mainly localized in anther tissues or microspores. Therefore, any putative rice candidate is likely to show a similar expression pattern to the *Arabidopsis* ortholog (Figure 4). Recently, a new rice resource was launch financed by the Ministry of Agriculture of Japan, RiceXPro (Rice Expression Profile Database). This is a repository of rice microarray transcriptome data, aimed at characterizing the expression patterns of all rice genes for functional genomic analysis. The expression profiles of putative rice orthologs can be confirmed using this service and then the gramene ortholog database can be subsequently used to confirm the expression pattern. Finally, synteny and phylogenetic analyses can be performed to establish the best candidates for further investigation.

Characterizing orthologous genes within different species requires a number of different bioinformatic analyses to confirm gene equivalence and functional conservation. Figure 4 shows some of the analysis required to accurately assign gene similarities. However, once the genes have been selected as putative orthologs, they need to be fully confirmed by mutant analysis, silencing or by complementing the *Arabidopsis* mutants. This is being facilitated by the availability of new tools that enable the analysis and characterization of putative orthologs in wheat and barley.

### Ortholog‐gene function confirmation

Ortholog localization using bioinformatics tools has improved greatly; however, to ultimately confirm orthology and functional conservation among species, further analysis is needed. Two main analyses can be conducted, complementary analysis and functional characterization within the species where the orthologs have been localized.

#### Complementary analysis

Complementary analysis is an essential tool for translational research and an effective way to confirm orthology. It consists of transforming the corresponding *Arabidopsis* heterozygous mutant with the orthologous gene and segregation analysis of complementation by the transgene. The use of heterozygotes and segregation analysis is necessary in lines with impaired fertility, since floral‐dipping transformation of homozygous male sterile lines is not possible. The putative ortholog needs to be fused to a promoter to drive its expression in the *Arabidopsis* mutant, either by a constitutive, overexpression promoter, or via the *Arabidopsis* native gene‐specific promoter. Although the first option is usually quicker, the results are not always satisfactory, due to the temporal and cell specific regulation observed in some genes. For instance, anther and pollen transcription factors such as *AtMS1* orthologs in rice and barley did not recover *Arabidopsis ms1* mutant fertility when driven by the CaMV35S overexpression promoter (Li et al. 2011; Fernández Gómez and Wilson 2014). However, once the rice and barley ortholog genes were fused to the *Arabidopsis AtMS1* native promoter, fertility was restored in the *ms1* homozygous mutant.

#### Functional characterization of ortholog genes

In order to characterize the ortholog genes of interest within the species of interest, rice, barley or wheat, a number of techniques are available. Mutant populations are available in rice, and the TILLING population in barley (Gottwald et al. 2009; Kurowska et al. 2011) and wheat (Chen et al. 2012) have contributed significantly to the characterization of genes and their networks (Wilson and Zhang 2009). In addition, highly efficient rice and barley *Agrobacterium*‐mediated transformation are now available (Nishimura et al. 2007; Harwood et al. 2009). Transformation approaches allow several ways to characterize the genes of interest. RNAi silencing is a useful technique to characterize gene function; however, this approach may not generate clear phenotypes due to the threshold level needed for effective silencing (Lindbo et al. 1993). RNAi target genes generally have reduced expression rather than being fully silenced (Yin et al. 2001), thus sufficient transcript may remain to maintain wild type function. This partial reduction in gene expression was seen in *HvMS1RNAi*silencing (Fernández Gómez and Wilson 2014), where pollen development was affected by the silencing and showed a partial sterile phenotype. In addition, RNAi silencing has been shown to be unreliable after successive generations. RNAi silencing construct consists of sense and antisense orientated DNA fragments separated by an intron. It has been observed that the antisense fragment tends to be lost, thus hairpin formation and failure of silencing is seen in subsequent generations (Watson et al. 2005).

Silencing approaches have been greatly improved by the use of the Chimeric Repressor gene‐Silencing Technology (CRES‐T). CRES‐T is a unique gene‐silencing method using plant‐specific chimeric transcriptional repressors. These chimeric repressors are produced from transcription factors by fusion with a transcriptional repression domain SRDX (Hiratsu et al. 2002) converting many transcription factors into strong transcriptional repressors. Chimeric repressors dominantly suppress target gene expression and thus confer loss‐of function phenotypes at high frequency, even in the case of functionally redundant transcriptional activators, in *Arabidopsis* and rice (Hiratsu et al. 2003). Therefore, this silencing technology offers a valuable tool to characterize genes and study gene networks in crops.

## CONCLUSIONS AND PROSPECTS

A high level of conservation is observed between pollen and anther development in monocots and dicots. This has been effectively illustrated in *Arabidopsis* and rice, but is also evident for temperate grasses and cereals, such as *Brachypodium*, wheat and barley. The lack of genome assemblies and genetic tools has limited translational approaches to the larger genomes of the temperate cereal crops. Nevertheless these are becoming more available and thus such analyses are becoming increasingly tractable. The increased number of genomes available is also aiding effective comparative analysis. These developments will enhance both understanding of the fundamental biology of crop species, but also will enable deployment of such traits for crop improvement.
